# Knowledge Gaps in Patients Undergoing Thyroid Fine-Needle Aspiration Biopsy

**DOI:** 10.2174/0118715303364657250415115259

**Published:** 2025-05-06

**Authors:** Mustafa C. Şenoymak, Nisa Babacanlar, Nuriye H. Erbatur, Ferrat Deniz, Arif Yönem

**Affiliations:** 1 Department of Endocrinology and Metabolism, University of Health Sciences, Sultan Abdulhamid Han Training and Research Hospital, Istanbul, Turkey

**Keywords:** Thyroid nodule, malignancy risk, fine-needle aspiration biopsy, informed consent, knowledge, health literacy

## Abstract

**Background:**

Fine-Needle Aspiration Biopsy (FNAB) is the most accurate diagnostic method to assess the malignancy risk of thyroid nodules. This study aims to assess the level of knowledge among patients regarding thyroid FNAB and its outcomes and to explore how this knowledge correlates with their sociodemographic and clinical characteristics.

**Methods:**

In a cross-sectional study at a tertiary healthcare facility's endocrinology and metabolism outpatient clinic, participants who had undergone their first thyroid FNAB and attended the clinic to review their results were included. Data collection encompassed demographic information, clinical history, and sonographic features of thyroid nodules. Patient knowledge was assessed using a 6-item questionnaire that evaluated awareness of the indications for thyroid FNAB, potential diagnostic outcomes, and the likelihood of a repeat biopsy.

**Results:**

A total of 423 patients participated in this study, with the majority being female (83.7%). The median age was 54 years. The indication for the biopsy was correctly identified by 68.5% of participants. Awareness of the potential for benign (85.1%) and malignant (84.6%) results was high. However, only 20.4% were informed about non-diagnostic outcomes and 16.6% about indeterminate results. Furthermore, 34.1% understood the need for a repeat biopsy. Participants under 65 years of age demonstrated significantly higher knowledge regarding the reason for the biopsy and the potential for benign or malignant results (*p* < 0.001). Participants with at least a high school education (38.1%) were more knowledgeable about all aspects of FNAB compared to those with lower educational levels (*p* < 0.05). Patients with a history of non-thyroidal malignancy (5.7%) demonstrated significantly greater understanding of non-diagnostic and indeterminate results (*p* < 0.001).

**Conclusion:**

The study reveals substantial knowledge gaps, particularly in understanding thyroid FNAB and its outcomes. Thus, targeted educational interventions are necessary to enhance patient comprehension and improve clinical outcomes.

## 1 INTRODUCTION

Thyroid nodules are a common clinical finding, and thyroid Fine-Needle Aspiration Biopsy (FNAB) is the gold standard diagnostic method for evaluating the malignant potential of thyroid nodules [1, 2]. This procedure is typically performed on patients who have been classified as at-risk based on their sonographic features. The cytology results from FNAB are categorized using the Bethesda classification system, which includes non-diagnostic, benign, indeterminate, and malignant categories [3]. These classifications are crucial, as they guide the follow-up and treatment plans for patients [1, 2].

The rate of Bethesda Category 1, representing non-diagnostic results, varies by biopsy center but is approximately 13% [4, 5]. This category indicates an insufficient number of thyroid cells for a definitive diagnosis, necessitating a repeat biopsy [1]. Bethesda Categories 3 and 4 are classified as indeterminate results. Although most nodules in these categories are benign, they may exhibit features suggestive of malignancy, such as nuclear atypia and architectural distortion, posing challenges for clinicians. Approximately 20% of FNABs result in indeterminate cytology [6-8]. For patients in these categories, molecular cytogenetic methods or a repeat biopsy are recommended for further risk assessment. In cases of recurrent non-diagnostic or indeterminate results, surgical intervention may be indicated [2, 9, 10].

Despite the widespread use of thyroid FNAB, patient understanding of the procedure and its outcomes remains suboptimal, which can impact adherence to follow-up care and overall clinical outcomes [11]. As with any medical procedure, patients should be thoroughly informed about thyroid FNAB and its potential outcomes before undergoing the procedure. Obtaining written consent is essential, as patients need to understand the indications for FNAB, the procedural steps, the implications of the results, and the subsequent actions required based on those results. While most patients primarily focus on whether their thyroid nodule is benign or malignant, understanding the possibility of non-diagnostic and indeterminate results is equally important for managing expectations and ensuring adherence to follow-up care. This level of knowledge is critical not only for improving patient compliance and understanding treatment outcomes but also for supporting informed decision-making in future healthcare scenarios [12-14]. Recent studies have revealed that patients often lack detailed knowledge about their medical procedures, not only in the context of thyroid FNAB but also across a wide range of interventional practices, including biopsies, surgeries, clinical research, and other medical interventions, despite having provided formal consent [11, 15, 16]. Moreover, a growing body of evidence indicates that improved patient understanding of their health conditions is strongly associated with better clinical outcomes [17-19]. Conversely, limited health literacy has been consistently linked to poorer outcomes across various medical conditions, including surgical interventions and chronic diseases [20, 21]. These findings underscore the critical importance of implementing targeted educational interventions to enhance patient comprehension and optimize healthcare delivery [22-24].

The aim of this study is to assess the level of knowledge among patients at a tertiary healthcare facility regarding thyroid FNAB and its potential outcomes. Additionally, this study seeks to explore the relationship between patients' knowledge levels and their sociodemographic and clinical characteristics, with a focus on identifying actionable strategies to improve patient education.

## 2 MATERIALS AND METHODS

### 2.1 Patients and Study Design

A cross-sectional study was carried out at the Department of Endocrinology and Metabolism, Sultan 2. Abdülhamid Han Training and Research Hospital, from September 2023 to July 2024. The study included patients who presented to the outpatient clinic with thyroid FNAB results, regardless of the requesting or performing clinician or the center where the procedure was conducted. The inclusion criteria consisted of individuals aged 18 years or older who had undergone FNAB for the first time and sought the center to review their results. Only patients who voluntarily consented to participate were included in the study. Written informed consent was obtained from all participants prior to their inclusion in the study. Patients were excluded from the study if they had previously undergone FNAB, had a history of thyroid malignancy, were under 18 years of age, or had an intellectual disability. Patients with prior FNAB experiences were excluded to eliminate potential bias arising from previous exposure to FNAB-related information, which could have influenced their responses and distorted the study’s findings on first-time patients' knowledge levels. Additionally, patients were excluded if they did not complete a written informed consent form prior to the FNAB procedure or if they underwent rapid on-site cytopathological evaluation.

Patient data collected included age, gender, educational background, history of hypothyroidism or hyperthyroidism, history of malignancy, sonographic features as classified by EU-TIRADS (European Thyroid Imaging Reporting and Data System), and nodule size [25]. Subsequently, a 6-item questionnaire was administered to assess patients' knowledge about the indications, potential outcomes of thyroid FNAB, and the necessity or likelihood of rebiopsy. The questionnaire was conducted in Turkish, verbally administered, and completed by the conducting researcher. The questions were adapted and translated into Turkish, with modifications, from the study conducted by Ospina *et al*., following formal permission obtained from the original authors. Given the absence of a validated questionnaire specific to this topic, the adapted tool was designed to address the study’s objectives effectively [11].

The first question, open-ended, was: “Do you know why you underwent thyroid fine-needle aspiration biopsy?” This question aimed to evaluate whether patients understood the reason for the biopsy. Patients who clearly identified that the procedure was performed to rule out malignancy in the thyroid nodule were considered knowledgeable.

The next four questions were structured as yes-or-no queries to assess knowledge about potential biopsy outcomes: “Do you know that the biopsy results could indicate benign results?”, “Do you know that the biopsy results could indicate malignant results?”, “Do you know that the biopsy results could indicate non-diagnostic results?”, and “Do you know that the biopsy results could indicate indeterminate results?”. The sixth and final question, also a yes-or-no query, was: “Would you need to undergo a repeat biopsy based on the results?” This question aimed to gauge patients' awareness regarding the need for a rebiopsy. Patients were classified as “knowledgeable” if they correctly answered specific questions related to FNAB potential outcomes and the necessity for repeat biopsies.

The study was approved by the Health Sciences University Hamidiye Scientific Research Ethics Committee (approval number 2023/16/26) and conducted in accordance with the Declaration of Helsinki.

### 2.2 Statistical Analyses

Statistical analyses for the study were conducted using SPSS v29. Descriptive data were presented as counts and percentages for categorical variables and as medians with Interquartile Ranges (IQR) for continuous variables. For comparisons between groups of categorical variables, Chi-Square and Fisher's Exact tests were used, depending on the distribution size. A significance level of 0.05 was accepted for all analyses. The required sample size was calculated using statistical methods to ensure adequate power for the study. Assuming an alpha level of 0.05, a power of 80%, and an expected effect size based on previous studies, we estimated that a minimum of 133 participants would be required to detect significant differences. However, to account for potential dropouts or incomplete data, a total of 423 patients were ultimately included in the study.

## 3 RESULTS

A total of 423 patients participated in the study, with the majority being female (83.7%, n=354). The median age of the participants was 54 years (IQR 16). Regarding educational background, 62.0% (n=262) of participants had an education level below high school, while 38.0% (n=161) had completed at least high school. Among the participants, 5.7% (n=24) had a history of non-thyroidal malignancy, 18.2% (n=77) had hypothyroidism, and 20.6% (n=87) had hyperthyroidism. Other characteristics, including the detailed educational background of patients, the sonographic classification of thyroid nodules using the EU-TIRADS system, and the size distribution of thyroid nodules, are presented in Table **1**.

When asked about the reason for undergoing the thyroid FNAB, 68.5% (n=290) of the participants correctly identified the purpose of the biopsy. Regarding the possible outcomes of the biopsy, 85.1% (n=360) were aware that the results could indicate a benign nodule, 84.6% (n=358) were aware of the potential for malignant results, 20.4% (n=87) were informed about non-diagnostic results, and 16.6% (n=71) recognized the possibility of indeterminate results. Additionally, 34.1% (n=144) of participants understood that a repeat biopsy might be necessary depending on the results (Table **2**).

When examining the distribution of participants' knowledge about the biopsy procedure based on their age, it was observed that a larger proportion of individuals under the age of 65 were informed across all aspects. The differences in the distribution of knowledge regarding the reason for the biopsy and the awareness that the results could be benign or malignant were statistically significant (Chi-Square Test, *p* < 0.001 for all three) (Table **3**).

Educational background significantly influenced patients' knowledge regarding FNAB. Participants with at least a high school education (n=161) demonstrated a greater understanding of FNAB compared to those with lower educational attainment (n=262). The percentages of patients correctly answering the knowledge-related questions based on their educational background are presented in detail in Table **4**.

Patients with a history of non-thyroidal malignancy (n=24) were more aware of the non-diagnostic and indeterminate results compared to those without malignancy (n=399) (*p* < 0.001). However, there was no significant difference in awareness of potential benign and malignant outcomes between these groups (*p* = 0.231 and *p* = 0.557, respectively) (Table **5**).

## 4 DISCUSSION

This study, pioneering in assessing thyroid fine-needle aspiration biopsy among the Turkish population, features a notably larger cohort from various centers, significantly enhancing the generalizability and representativeness of the findings compared to previous studies in this field. The findings reveal a significant lack of knowledge among patients regarding thyroid FNAB, emphasizing a critical need for enhanced patient education and counseling. By identifying specific knowledge gaps and exploring the influence of sociodemographic and clinical factors on patient understanding, this study contributes to the growing body of literature on health literacy and patient-centered care. These insights can inform the development of targeted educational interventions and standardized counseling protocols to improve adherence and healthcare outcomes.

Comparative analyses reveal that the extent of patient knowledge on thyroid FNAB remains limited. A landmark study by Ospina *et al*. at the Mayo Clinic, which included 196 patients, indicated that while a majority were aware of FNAB indications (86%) and potential benign or malignant results (90%), understanding of non-diagnostic (71%) and indeterminate results (68%) was less common, with only 31% recognizing the need for potential repeat biopsies [11]. In contrast, our findings show a slightly higher awareness of the necessity for repeat biopsies but generally lower knowledge levels regarding other FNAB outcomes. This variation can largely be attributed to the educational disparities between the cohorts—our study predominantly involved patients with education levels below high school, unlike the higher educational levels observed in the study of Ospina *et al*. Furthermore, differences in healthcare policies and practices across countries may have contributed to these discrepancies in patient understanding. To elaborate further, variations in the consent process, such as differences in how informed consent is obtained, the duration of patient consultations, and the time interval between the initial consultation and the procedure, may also have influenced these outcomes. For instance, shorter consultation times or delays between the recommendation and execution of the biopsy could limit the opportunity for patients to fully comprehend the procedure and its potential outcomes.

The pattern of limited patient knowledge extends beyond thyroid biopsies to other invasive procedures as well [16]. For example, a study conducted in China on endoscopic ultrasound-guided biopsies revealed that only a minor fraction of patients comprehended the potential for non-diagnostic or indeterminate results, even though they had provided written consent beforehand [15]. A study conducted in the emergency department revealed that 78% of patients demonstrated deficient comprehension of their care and discharge instructions, with most patients unaware of their lack of understanding [26]. Rosique *et al*. demonstrated in their study that two-thirds of patients were unable to recall the information provided to them by the anesthesiologist, highlighting significant gaps in the communication and retention of critical medical details [27]. These observations underscore a widespread deficiency in patient understanding across various populations and medical procedures, highlighting a universal challenge in healthcare communication and education.

A significant barrier to patient comprehension of interventional procedures is clinicians' tendency to provide insufficiently detailed explanations. The variability in communication styles among healthcare professionals, along with the limited time allocated for patient consultations, further exacerbates this issue [28, 29]. The physician performing the biopsy is not always the same one who initially recommended it, which can disrupt effective communication and continuity of care [30]. Additionally, the varying intellectual levels of patients represent another critical variable that may contribute to these challenges [16, 31]. These issues are compounded by a generally insufficient focus on patient-centered decision-making within the healthcare community [32]. Efforts to improve patient understanding should include enhancing patient-physician communication, ensuring adequate examination time, and utilizing visual aids like video explanations, which have proven to be effective [33-35]. Streamlining the process from biopsy recommendation to execution, offering comprehensive explanations during consent, addressing inquiries thoroughly, and involving family members are crucial for fostering informed decision-making and better engagement [36-39]. Some other potential recommendations include tailoring communication methods to align with each patient's intellectual capacity, providing printed educational materials for patients to review at their own pace, and verifying comprehension through teach-back methods to ensure that the information has been clearly understood [16, 40].

Obtaining written informed consent is a cornerstone of standard practice prior to invasive procedures like FNAB. Although there is no standardized form across all countries, they generally inform patients about the clinician performing the procedure, the reasons for the procedure, potential complications, and expected outcomes [41, 42]. For instance, the one used at our center addresses all questions from our study's survey. Nevertheless, our findings, along with others, suggest that the inclusion of detailed information in consent forms does not guarantee patient comprehension [11, 15, 43]. A critical review found that adequate overall understanding of the information provided and the risks associated with surgery was demonstrated in only 29% and 36% of studies providing relevant data, respectively [43]. This gap in knowledge suggests that patients often perceive consent as merely a procedural formality rather than a means to gain a comprehensive understanding. An important finding in this context comes from the study by Rosique *et al*., which demonstrated that 21% of patients who signed a required consent form for anesthesia did not read it, highlighting that obtaining formal consent alone may not suffice to ensure thorough patient comprehension [27].

Our study found that individuals with higher education levels demonstrated greater knowledge and higher accuracy in answering nearly all questions, reinforcing the well-established correlation between educational attainment and health-related knowledge [44-47]. This highlights the influence of educational attainment on health literacy and suggests that targeted educational programs tailored to less-educated individuals may help bridge the knowledge gap. It is crucial to support formal education and integrate healthcare topics and research skills into curricula to enhance public health literacy and optimize patient education [48]. Prior studies have shown that increasing health literacy is associated with better outcomes in numerous clinical scenarios, including surgical interventions and chronic conditions. A review of 48 studies by Billany and colleagues highlights that higher health literacy among patients with chronic kidney disease consistently correlates with improved self-management behaviours, suggesting that better outcomes may be associated with this [49]. Conversely, limited health literacy has been linked to higher mortality rates, increased hospitalizations, and poorer clinical outcomes, all of which contribute to higher overall healthcare expenses [22-24, 49, 50]. These findings underscore the critical need for targeted educational interventions to improve health literacy and empower patients across diverse healthcare settings.

Improving patient education and communication regarding thyroid FNAB is crucial for several reasons. Knowledge gaps regarding FNAB outcomes, such as non-diagnostic and indeterminate results, may lead to delayed follow-up and suboptimal treatment adherence. Well-informed patients are more likely to engage actively in shared decision-making, leading to increased satisfaction and adherence to treatment plans [48, 51]. In the study by Wu *et al*., it is emphasized that properly informing the patient significantly enhances shared decision-making between patients and surgeons, particularly in the context of using intraoperative neural monitoring during thyroid and parathyroid surgery, thereby promoting collaborative management of the patient's treatment [51]. Moreover, addressing knowledge gaps and enhancing patient understanding can help alleviate anxiety and concerns related to FNAB procedures and outcomes [35, 36, 39, 52]. For instance, in the study by Ay and colleagues, patients who were informed through video materials about thyroid FNAB exhibited lower anxiety levels during the biopsy procedure [35].

Our findings reveal that patients with a history of non-thyroidal malignancy were notably more knowledgeable about the FNAB procedure. This increased awareness can likely be attributed to their prior experiences within healthcare settings, which might have involved similar diagnostic processes or required them to engage actively with medical information. Such patients may have had repeated interactions with healthcare providers about the implications of diagnostic tests, which could enhance their understanding of medical procedures in general. Furthermore, these patients are often more exposed to the necessity of understanding medical information to make informed decisions about their treatment options [46, 53-55].

Our study found that patients under the age of 65 demonstrated significantly higher knowledge levels regarding thyroid FNAB compared to those aged 65 and above. This finding aligns with previous research indicating that younger patients tend to have better health literacy and more active engagement with health-related information. The lower knowledge levels among older patients may be attributed to several factors, including lower educational attainment, limited access to digital health resources, and cognitive decline associated with aging [56-58]. These findings underscore the need for tailored educational interventions for older adults, such as simplified educational materials, one-on-one counseling sessions, and the involvement of family members in the education process [59, 60].

Our study has certain limitations. We did not investigate the time interval between the provision of information about the procedure and its actual execution, nor did we examine the duration of patient examinations—both of which could have influenced the results. The study also did not analyze differences based on the center where the procedure was performed or the varying expertise and backgrounds of the clinicians involved. This lack of differentiation may have obscured important individual variations. Additionally, administering the questionnaire immediately after the FNAB, rather than during the follow-up visit when patients received their results, might have influenced their responses.

## CONCLUSION

This study underscores the importance of assessing the knowledge of patients about thyroid-fine needle aspiration biopsy. Comprehensive counseling is not only essential for ensuring that patients are well-informed about the procedure, its potential outcomes, and the possibility of a rebiopsy, but also for addressing the specific needs of individuals with limited educational backgrounds. Enhancing patient education and communication can empower patients, promote informed decision-making, and ultimately improve the overall quality of care in the evaluation of thyroid nodules. Moreover, targeted educational interventions, such as simplified consent forms, visual aids, and structured counseling sessions, are necessary to improve patient understanding and adherence to follow-up care.

## Figures and Tables

**Table 1:** Demographic and clinical characteristics of study participants.

**Gender**	**Number (%)**
Female	354 (83.7)
Male	69 (16.3)
Educational Background	
Literate	21 (5.0)
Primary School	205 (48.5)
Secondary School	36 (8.5)
High School	79 (18.7)
University	82 (19.4)
Medical History	
Non-Thyroidal Malignancy	24 (5.7)
Hypothyroidism	77 (18.2)
Hyperthyroidism	87 (20.6)
EU-TIRADS Score	
3	165 (39.0)
4	139 (32.9)
5	119 (28.1)
Nodule Size	
0-9 mm	6 (1.4)
10-19 mm	209 (49.4)
20-29 mm	128 (30.3)
30-39 mm	51 (12.1)
>40 mm	29 (6.9)

**Abbreviation:** EU-TIRADS: European Thyroid Imaging Reporting and Data System.

**Table 2:** Patients’ knowledge regarding thyroid fine-needle aspiration biopsy and ıts outcomes.

-	**Number (%)**
Aware of the reason for the biopsy	290 (68.5)
Aware that the result can be benign	360 (85.1)
Aware that the result can be malignant	358 (84.6)
Aware that the result can be non-diagnostic	87 (20.4)
Aware that the result can be indeterminate	71 (16.6)
Aware that the procedure might need to be repeated	144 (34.1)

**Table 3:** Evaluation of patient knowledge regarding FNAB based on age.

**Age**	**≥ 65** **(n=87)** **n (%)**	**<65** **(n=336)** **n (%)**	*p* value**
Aware of the reason for the biopsy	44 (50.6)	246 (73.2)	<0.001
Aware that the result can be benign	64 (73.6)	296 (88.1)	<0.001
Aware that the result can be malignant	63 (72.4)	295 (87.8)	<0.001
Aware that the result can be non-diagnostic	16 (18.4)	71 (21.1)	<0.573
Aware that the result can be indeterminate	12 (13.8)	59 (17.6)	<0.402
Aware that the procedure might need to be repeated	27 (31.0)	118 (35.1)	<0.474

**Note:** FNAB: Fine Needle Aspiration Biopsy, n: number **Chi-SquareTest, A *p*-value less than 0.05 is considered statistically significant.

**Table 4:** Evaluation of patient knowledge regarding FNAB based on educational background.

**Educational Background**	**Secondary School or Lower** **(n=262)** **n (%)**	**High School or Higher** **(n=161)** **n (%)**	*p* value**
Aware of the reason for the biopsy	140 (53.4)	150 (93.2)	<0.001
Aware that the result can be benign	203 (77.5)	157 (97.5)	<0.001
Aware that the result can be malignant	202 (77.1)	156 (96.9)	<0.001
Aware that the result can be non-diagnostic	26 (9.9)	61 (37.9)	<0.001
Aware that the result can be indeterminate	22 (8.4)	49 (30.4)	<0.001
Aware that the procedure might need to be repeated	54 (20.6)	91 (56.5)	<0.001

**Note:** FNAB: Fine Needle Aspiration Biopsy, n: number **Chi-SquareTest, A *p*-value less than 0.05 is considered statistically significant.

**Table 5:** Evaluation of patient knowledge regarding FNAB based on history of non-thyroidal malignancy.

**History of Non-Thyroidal Malignancy**	**Yes** **(n=24)** **n (%)**	**No** **(n=399)** **n (%)**	*p*-value*
Aware of the reason for the biopsy	21 (87.5)	269 (67.4)	0.042
Aware that the result can be benign	23 (95.8)	337 (84.5)	0.231
Aware that the result can be malignant	22 (91.7)	336 (84.2)	0.557
Aware that the result can be non-diagnostic	14 (58.3)	73 (18.3)	<0.001
Aware that the result can be indeterminate	12 (50.0)	59 (14.8)	<0.001
Aware that the procedure might need to be repeated	19 (79.2)	126 (31.6)	<0.001

**Note:** FNAB: fine Needle Aspiration Biopsy, n: number, A *p*-value less than 0.05 is considered statistically significant *Fisher Exact Test.

## Data Availability

The data that support the findings of this study are available from the corresponding author upon reasonable request.

## LIST OF ABBREVIATIONS

IQR: Interquartile Ranges
EU-TIRADS: European Thyroid Imaging Reporting and Data System
FNAB: Fine-needle Aspiration Biopsy

## STANDARDS OF REPORTING

STROBE guidelines were followed.
